# Function Preservation of the Upper Lip after Tumor Resection Using Residual Orbicularis Oris Muscle and Attached Levator Labii Superioris Alaeque Nasi

**DOI:** 10.1097/GOX.0000000000001962

**Published:** 2018-10-04

**Authors:** Masamitsu Kuwahara, Satoshi Yurugi, Chikako Sasaki, Takashi Nakanishi, Mika Takeuchi, Riyo Miyata, Masayuki Harada, Yasumitsu Masuda

**Affiliations:** From the Division of Plastic Surgery, Nara Medical University Hospital, Nara, Japan.

## Abstract

We report a case, function preservation of the upper lip after tumor resection was possible using residual orbicularis oris muscle and attached levator labii superioris alaeque nasi. Patient was 67-year-old male with squamous cell carcinoma at the vermilion border. The tumor was resected with an 8-mm margin, leaving the oral mucosa as intact as possible. To reconstruct the red lip, we used the oral mucosa as a rotational transposition flap. The white lip was reconstructed with a cheek rotation flap. A levator labii superioris alaque nasi muscle flap, which was attached to the remaining orbicularis oris muscle, was used to increase marginal lip volume. The movement of the reconstructed lip was good. At 9 postoperative months, induration of the red lip was palpable, and we suspected that the blood supply to the levator labii superioris alaque nasi was borderline insufficient. Slight drooping of the reconstructed lip occurred. We dissected this was caused by dissection of mid facial muscles from orbicularis oris muscle to ease downward rotation of the cheek flap and obscure the original nasolabial fold. Although some drooping and induration of the lip occurred, the white and red lip were reconstructed in a single-stage procedure, which resulted in good movement and preserved the function of the orbicularis oris muscle.

Cross-lip flaps are the flaps that are most commonly used to repair full-thickness partial marginal lip defects in which the defect’s vertical dimension is longer than its horizontal dimension, although this method requires a 2-stage operation.^1^ Cross-lip flaps can be used to reconstruct red lip tissue while preserving orbicularis oris muscle function.

Treating a lip defect whose vertical dimension is shorter than its horizontal dimension with a cross-lip flap can result in microstomia.

Vermilion advancement flaps are useful for reconstructing relatively small marginal lip defects.^2^ However, in this method, Cupid’s bow sometimes has to be ignored when there is a paucity of lateral red lip tissue next to the defect.

We encountered a partial marginal lip defect, which would have been hard to treat with the abovementioned methods. Therefore, we reconstructed it by using the remaining orbicularis oris muscle, the levator labii superioris alaeque nasi (LLSAN) muscle, and a cheek rotation flap.

The patient was a 67-year-old male. He was diagnosed with squamous cell carcinoma based on a biopsy examination of the vermilion boarder of the upper lip. We planned to resect the tumor together with an 8-mm safety margin (Fig. 1).

**Fig. 1.**
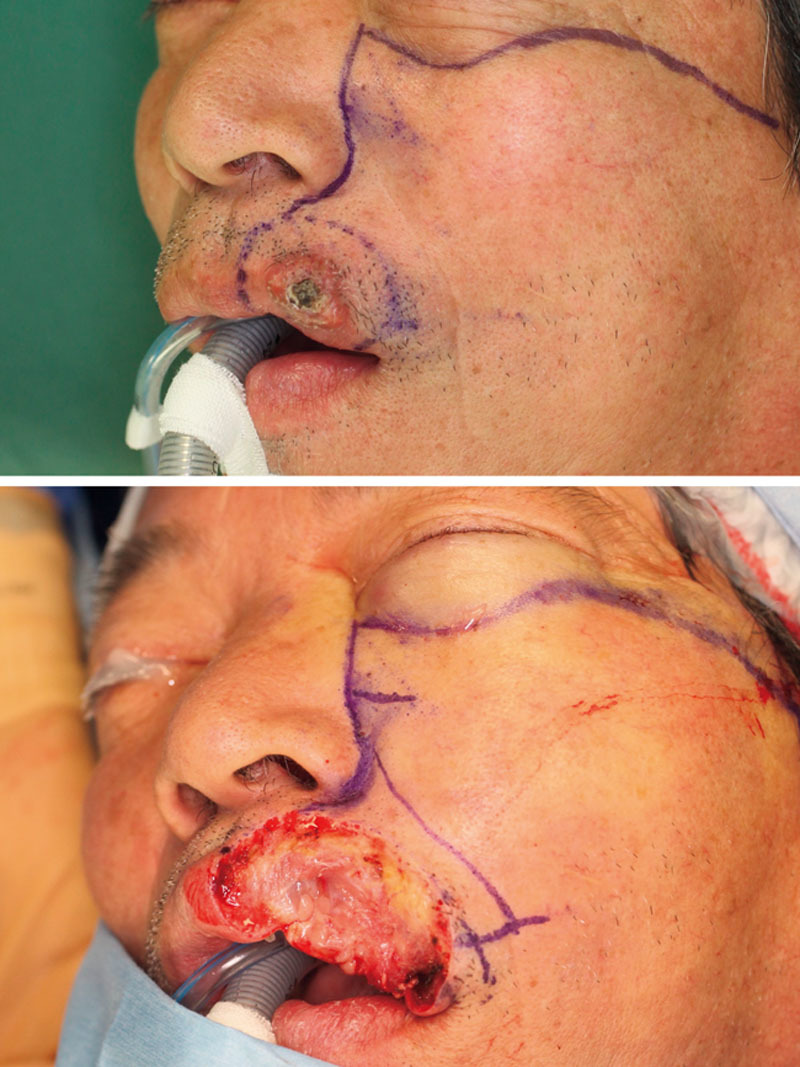
Squamous cell carcinoma at the vermilion border of the upper lip was resected together with an 8-mm safety margin, leaving the oral mucosa as intact as possible.

We resected the tumor together with most of the orbicularis oris muscle under the resection line, but left the oral mucosa as intact as possible.

After the tumor had been resected, we designed a cheek rotation flap for reconstructing the white upper lip tissue and then dissected the flap above the mid-facial muscles.

To reconstruct the red lip tissue, we used the oral mucosa as a rotational transposition flap. To augment the marginal lip defect, the LLSAN muscle was detached from the maxillary bone surface and raised as a muscle flap attached to the remaining portion of the orbicularis oris muscle (Fig. 2).

**Fig. 2.**
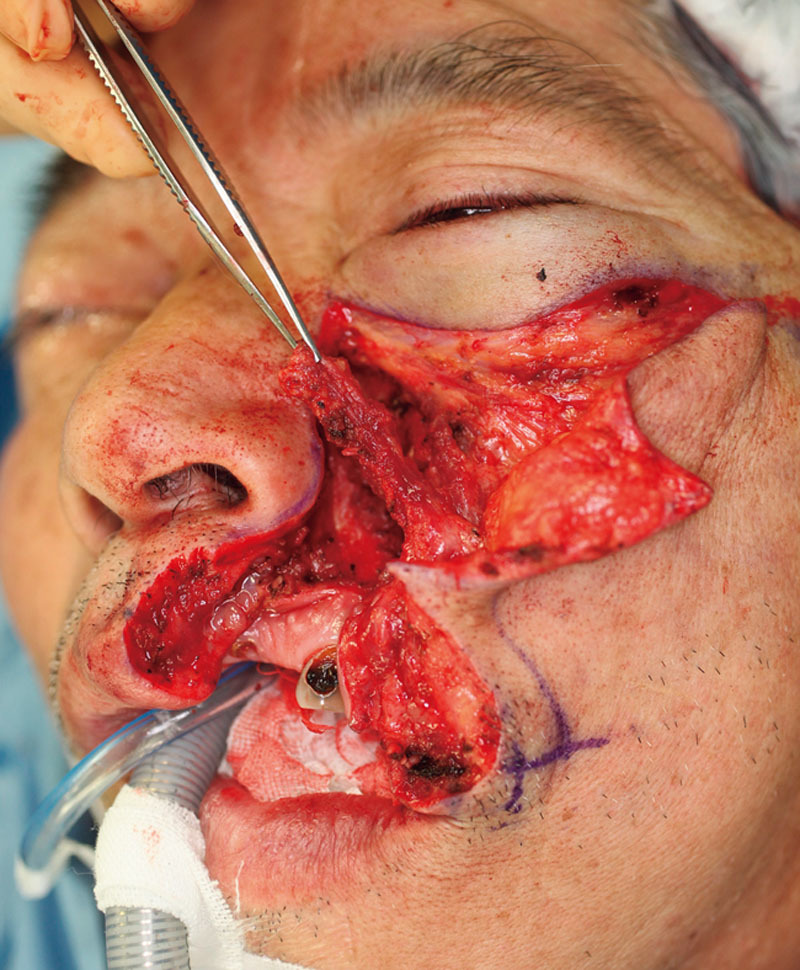
The LLSAN muscle was detached from the maxillary bone surface. It remained attached to the residual portion of the orbicularis oris muscle. The LLSAN muscle was transferred to augment the vermilion defect.

The levator labii superioris and zygomaticus minor muscles were partially bluntly dissected at their insertions and moved horizontally toward the previous position of the LLSAN. This dissection procedure allowed downward rotation of the cheek flap and the orbicularis oris muscle to reconstruct the white lip tissue and to obscure the newly positioned nasolabial fold. The cheek flap was large enough to cover the defect.

In this way, we were able to reconstruct both the white and red lip tissue, while preserving the function of the residual orbicularis oris muscle, in a 1-stage operation. For the first 3 months after surgery, the patient complained of drooling during drinking. The lower eyelid swelling caused by the procedure improved within 4 months.

The reconstructed lip exhibited good movement, but some induration was palpable under the red lip tissue; however, this improved within 9 months.

No tumor recurrence had been detected at 2 postoperative years, and there was no contracture of the reconstructed lip or mucosa.

Slight drooping of the reconstructed lip and the margin of Cupid’s bow occurred (Figs. 3, 4). The patient was satisfied with the results of the procedure and did not want to undergo further revision.

**Fig. 3.**
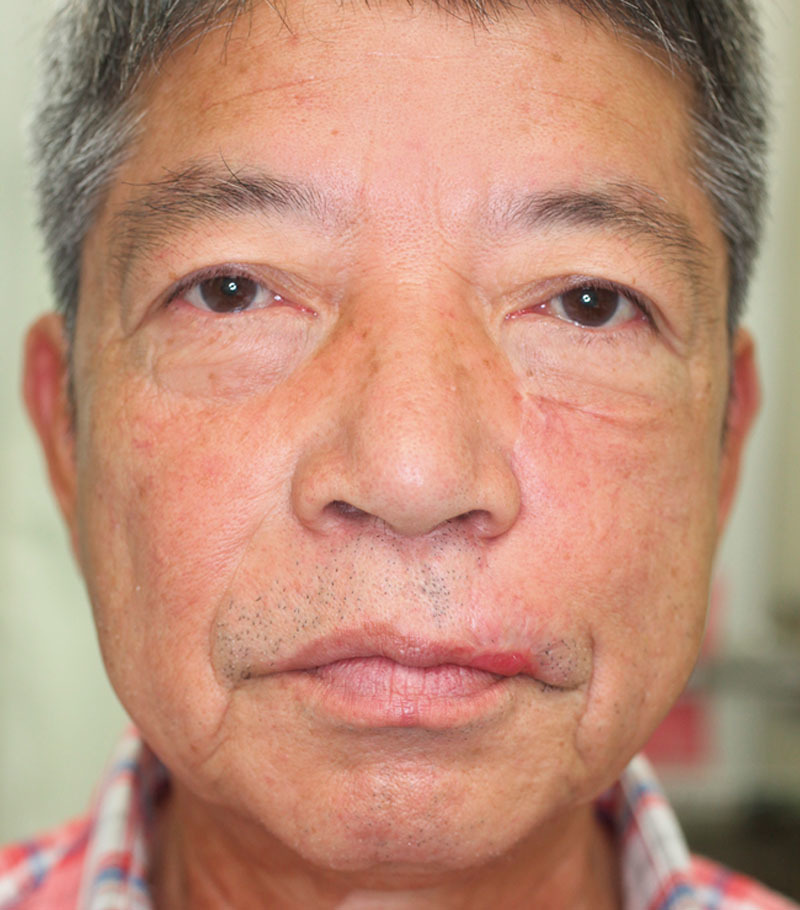
The patient’s appearance at 9 months after the 1-stage surgical procedure Slight drooping of the reconstructed lip and the margin of Cupid’s bow occurred.

**Fig. 4.**
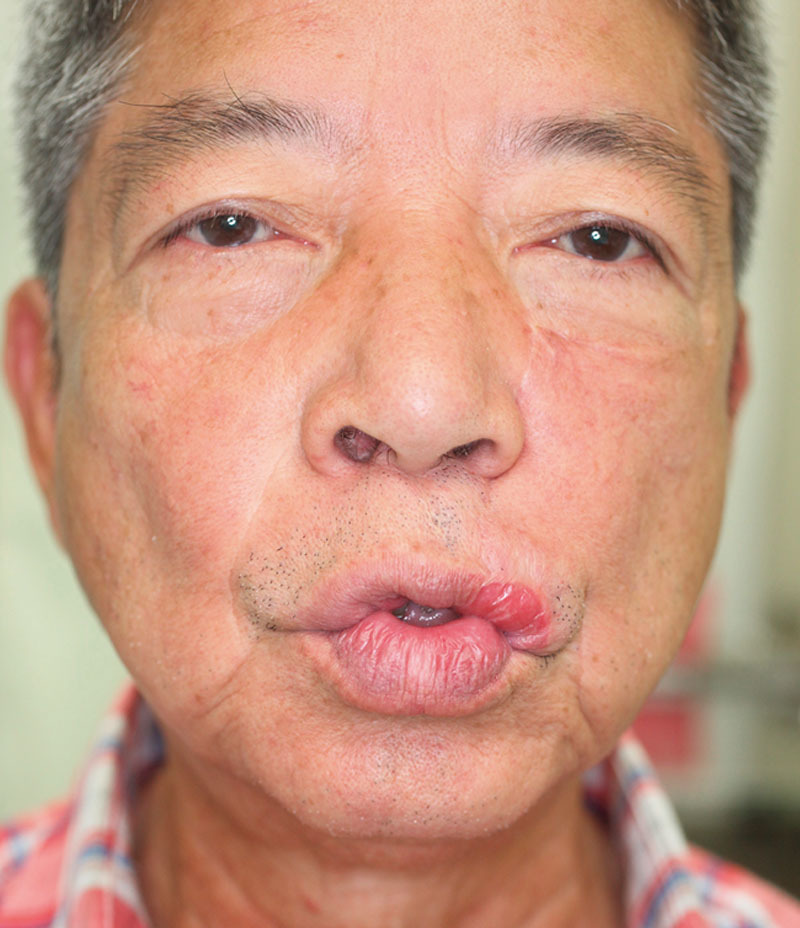
The reconstructed lip exhibited good movement. When the patient made a “WOO” sound, some heightening was seen at the margins of the reconstructed lip. We suspect that this was due to the effects of the procedure on the functioning of the orbicularis oris muscle.

## DISCUSSION

In the reconstruction procedure described in this report, a portion of the orbicularis oris muscle that is attached to the LLSAN must be preserved. In the current case, we were able to reconstruct the red lip tissue using a rotational transposition flap derived from the oral mucosa. This resulted in some horizontal shortening of the upper lip. If the mucosal defect had been larger, we would have had to choose another method to reconstruct the red lip tissue.

The LLSAN muscle originates from the maxillary processus frontalis, and its insertion is located between the levator anguli oris and orbicularis oris muscles.^3^ It pulls the upper lip and nasal alae. It has 2 bellies and merges with the nasalis levator labii superioris muscle at its insertion. Blood is supplied to it by the facial artery.

The use of the LLSAN to produce a musculocutaneous flap has been reported previously.^4^ The report stated that there were no blood perfusion issues in the 10 cases experienced by the authors. However, we consider that it is difficult to attach the entire facial artery to this muscle. Depending on the course of the facial artery and the size of the defect, there might be a risk of the blood supply to this muscle being poor, especially at its origin. At about 9 postoperative months, induration was palpable in the reconstructed red lip. Although we did not experience any reduction in volume, we suspect that the blood flow to the LLSAN was borderline insufficient. To increase the volume or blood flow of such flaps, it might be possible to include the levator labii superioris muscle.

Little drooping of the reconstructed lip was seen (Fig. 3). We dissected some of the levator labii superioris and zygomaticus minor muscles at their insertions at the orbicularis oris muscle to ease the horizontal movement and downward rotation of the cheek flap and to obscure the original nasolabial fold.

We were also able to obscure the novel nasolabial fold, but this resulted in a drooping lip.

When the patient made a “WOO” sound, some heightening was seen at the margins of the reconstructed lip (Fig. 4). We suspect that this was due to the effects of the procedure on the functioning of the orbicularis oris muscle.

In conclusion, in a patient with a partial upper-lip defect the white and red lip tissue were reconstructed in a 1-stage procedure, which resulted in the remaining orbicularis oris muscle exhibiting good movement, although some drooping and induration of the lip occurred.
